# A systematic, standard-based, participatory assessment of a Continuous Quality Improvement project in Kyrgyzstan and Tajikistan: results for maternal care

**DOI:** 10.7189/jogh.15.04176

**Published:** 2025-05-23

**Authors:** Tinatin Gagua, Dimitry Beglitse, Anna Calancae, Dilrabo Yunusova, Arsen Askerov, Zarina Ibragimova, Asel Orozalieva, Nurshaim Tilenbaeva, Shoira Yusupova, Oleg Kuzmenko, Sophie Jullien, Martin W Weber, Kyrgyzstan Tajikistan Quality of Maternal Care Quality Improvement Group

**Affiliations:** 1 Davit Tvildiani Medical University, Tbilisi, Georgia; 2 Medical institute named after S. I. Georgievsky of V. I. Vernadsky Crimean Federal University, Simferopol, Russia; 3 Mother and Child Institute, Chisinau, Republic of Moldova; 4 Department of Safe Motherhood and Family Planning, Ministry of Health and Social Protection of the population of Tajikistan, Dushanbe, Tajikistan; 5 Ministry of Health of the Kyrgyz Republic, Association of Obstetricians and Gynecologists of the Kyrgyz Republic, Bishkek, Kyrgyzstan; 6 Association of Midwives in Tajikistan, Dushanbe, Tajikistan; 7 Alliance of Midwives of the Kyrgyz Republic, Bishkek, Kyrgyzstan; 8 Quality of Care and Patient Safety Office, World Health Organization, Regional Office for Europe, Athens, Greece; 9 WHO Country Office of Tajikistan, Dushanbe, Tajikistan; 10 Sexual and Reproductive Health Division of Country Health Policies and Systems, World Health Organization, Regional Office for Europe, Copenhagen, Denmark

## Abstract

**Background:**

Maternal health care quality remains challenging in low- and middle-income countries, including Central Asia, where access to effective care is limited. While quality improvement (QI) interventions have been introduced, their impact is rarely evaluated. This study evaluates the effects of a two-year, complex QI intervention to improve maternal health services in Kyrgyzstan and Tajikistan.

**Methods:**

We employed a pre-post intervention design to evaluate improvements in maternal health care quality in 19 hospitals across Kyrgyzstan and Tajikistan. Following an initial assessment, an action plan was developed using a WHO-standardised tool. The study implemented a multi-faceted intervention to improve maternal health. A 0–3 scoring system measured changes over time. No control group was included.

**Results:**

In Kyrgyzstan, significant improvements were observed in caesarean section management (mean (x̄) = 1.9–2.1, *P* = 0.01), maternal complications management (x̄ = 1.6–1.9, *P* = 0.01), postpartum haemorrhage management (x̄ = 1.8–2.1, *P* = 0.03), and preeclampsia management (x̄ = 1.4–1.9, *P* = 0.01). Changes in hospital support services (x̄ = 1.6–1.8, *P* = 0.68) and infection control policies (x̄ = 1.6–1.9, *P* = 0.32) were not statistically significant. In Tajikistan, statistically significant improvements were seen in hospital support services (x̄ = 1.4–2.0, *P* = 0.01), routine labour and vaginal birth care (x̄ = 1.4–2.0, *P* = 0.01), infection control policies (x̄ = 1.4–1.8, *P* = 0.03), maternal complications management (x̄ = 1.5–2.1, *P* = 0.02), postpartum haemorrhage (x̄ = 1.7–2.1, *P* = 0.04), and labour progress (x̄ = 1.2–2.1, *P* = 0.01). However, changes in caesarean section management (x̄ = 1.7–2.3, *P* = 0.09) and emergency preparedness (x̄ = 1.6–2.3, *P* = 0.11) did not reach statistical significance.

**Conclusions:**

The WHO-guided participatory approach set benchmarks that improved labour management, obstetric care, infection control, and infrastructure. Expanding such initiatives, especially in underserved areas, is vital to sustain and scale their impact on maternal health.

High maternal and neonatal mortality and morbidity rates persist in many countries despite rising institutional birth rates. This underscore significant deficiencies in the quality of care and structural challenges within health systems [1]. Poor quality of care contributes to high levels of maternal and neonatal deaths [2,3]. Efforts to improve health care in low and low-middle-income countries have shifted from focusing on access to health care to enhancing health care quality and the health system. This aligns with the World Health Organization's (WHO) vision: ‘Every pregnant woman and newborn receives quality care throughout pregnancy, childbirth, and the postnatal period’ [4]. Childbirth-associated risks can be reduced when the facilities are well-equipped, and health workers are skilled and knowledgeable [5]. While quality improvement (QI) interventions are increasingly being implemented in low- and middle-income countries (LMICs), there is a need for more comprehensive evaluations of their impact. Many studies have reported the effectiveness of quality assessment tools in identifying priority areas [6]. However, the challenge lies in identifying feasible QI strategies with limited resources that are sustainable over time and cover all essential aspects of care. Maternal and neonatal health care in Central Asia, specifically in Tajikistan and Kyrgyzstan, continues to face significant challenges. Despite efforts to expand health care access, maternal and neonatal mortality rates remain high [7–9]. These outcomes are frequently attributed to critical gaps in the quality of care, inadequate health care infrastructure, and systemic barriers within health systems, including disparities in resource allocation and training of health care personnel [10]. These factors and logistical considerations made them suitable choices for implementing and evaluating quality improvement interventions. World Health Organization supported a 24-month QI project in Kyrgyzstan and Tajikistan in collaboration with the Ministry of Health. The main objective of this project was to improve the quality of maternal, newborn, and child care in Kyrgyzstan and Tajikistan by identifying key issues and implementing actions within the QI cycle. The objective of this study was to assess the impact of this project on improving maternal health care quality. More details on the countries and other aspects of the care for neonates and children are reported elsewhere [11–14].

## METHODS

We employed a pre- and post-mixed methods study approach. This included a baseline assessment, and an action plan was developed based on assessment results. We used a standard matrix provided by the WHO to guide the formulation of targeted interventions [15,16]. After implementing the QI interventions described elsewhere [12,14], we conducted an end-line assessment to evaluate progress and identify ongoing challenges. The same methodology was used in Kyrgyzstan and Tajikistan. Given the two-year study period, we focused on process indicators rather than long-term health outcomes, such as maternal and neonatal mortality requiring extended follow-up.

### Settings

In collaboration with the WHO Regional Offices, the Ministries of Health in Kyrgyzstan and Tajikistan selected nine and ten pilot district hospitals, respectively. The selection of facilities followed predefined criteria to ensure:

1) geographic coverage across at least three regions of provinces in each country, and

2) inclusion of different levels of the health system, with a minimum of one referral hospital selected per country.

### Assessment process

A baseline assessment was conducted using the revised 2021 Maternal and Newborn Hospital Quality Assessment and Improvement Tool (WHO Europe, 2014 edition) [15], along with the WHO Effective Perinatal Care training package and IMPAC recommendations [17]. The tool covers three areas: hospital support services, case management, and policy/service organisation. Data were collected through observations, records, statistics, and interviews with staff and mothers. A pre-post design combined quantitative (0–3 scale) and qualitative data from provider interviews. Each item was scored through triangulation of sources to ensure a comprehensive and reliable evaluation.

The 0–3 scoring system reflects care quality: 0 – very low quality, with harmful or absent care (*e.g*. inability to perform emergency CS); 1 – inadequate care with serious risks or rights violations (*e.g*. lack of evidence-based practices like steroid use in preterm labour); 2 – suboptimal but not dangerous care, with some rights respected; 3 – care aligned with international standards, requiring little to no improvement.

An international team of obstetrics, midwifery, neonatology experts, and national professionals selected by the Ministries of Health conducted the assessments. The teams were trained and calibrated to ensure consistent scoring, and senior supervisors oversaw quality.

In 2023, progress was evaluated using the same tool and independent teams, blinded to baseline data. This follow-up assessment, conducted under a standardised protocol, measured the impact of QI interventions across both countries.

### QI cycle in the facilities and the national process

Based on the assessment results, a 24-month action plan was developed for each hospital. The plan was created with hospital teams, incorporated input from health care workers and patients, and outlined priorities, responsibilities, and timelines. Key interventions included:

• Establishing QI teams and hospital-specific improvement plans.

• Holding semi-annual national quality improvement meetings and workshops for all staff, hospital directors, obstetricians, midwives, nurses, and neonatologists to discuss progress and challenges.

• Providing supportive supervision, capacity building, and ongoing guidance.

• Offering technical support for creating and updating guidelines.

• Providing perinatal care training to enhance clinical skills.

### Data analysis

Teams summarised mean scores by chapter and subchapter in Excel, using colour-coded heat charts. Differences were analysed following the approaches described by Yusupova et al. [12] and Tilenbaeva et al. [14]. The Wilcoxon signed-rank test (SPSS 27) assessed maternal care changes, chosen for paired, ordinal, non-normally distributed data, and small samples. Statistical significance was set at *P* < 0.05.

### Ethics approval

The Ministries of Health approved the project under WHO Europe and Biennial Agreements (Kyrgyzstan: orders #1392/2021, #1218/2023; Tajikistan: #118/2021, #688/2023). Hospital management endorsed the methods, and informed consent was obtained from all participants before the assessment.

## RESULTS

### Hospital support services

The baseline assessment revealed significant quality gaps in Kyrgyzstan and Tajikistan, particularly in infrastructure, personnel, and central supply systems. Physical infrastructure, staffing, essential services, supplies, ward conditions, and laboratory services scored below 2 (on a 0–3 scale).

#### Kyrgyzstan

At the end-line assessment, the mean score increased from 1.7 to 1.8 (*P* = 0.68). Pharmacy management and medical availability scores rose from 1.9 to 2 (*P* = 0.55). Equipment, supplies, and laboratory services showed improvement, with scores increasing from 1.4 to 1.8 (*P* = 0.06), and ward infrastructure improved from 1.7 to 1.8 (*P* = 0.89). However, physical infrastructure remained unchanged at 1.5, and statistics and health management decreased from 2.1 to 1.8 (*P* = 0.11).

#### Tajikistan

The baseline score of 1.2 improved to 1.9 (*P* = 0.01). Hospital support services showed universal improvement, with pharmacy, medical availability, and equipment scores rising from 1.0 and 1.1 to 2.0 (*P* = 0.05). Physical infrastructure, staffing, and basic services improved from 1.4 to 1.9 (*P* = 0.01), and data management systems increased from 1.4 to 2.2 (*P* = 0.01). Supplies, laboratory services, and ward infrastructure also improved, with scores rising from 1.0 to 1.8 (*P* = 0.01), 1.2 to 1.8 (*P* = 0.01), and 1.5 to 2.0 (*P* = 0.03).

Despite improvements, average scores in hospital support services in both countries remained below the acceptable threshold (<2.0).

### Routine care for labour and vaginal births

#### Kyrgyzstan

**Figure 1:**
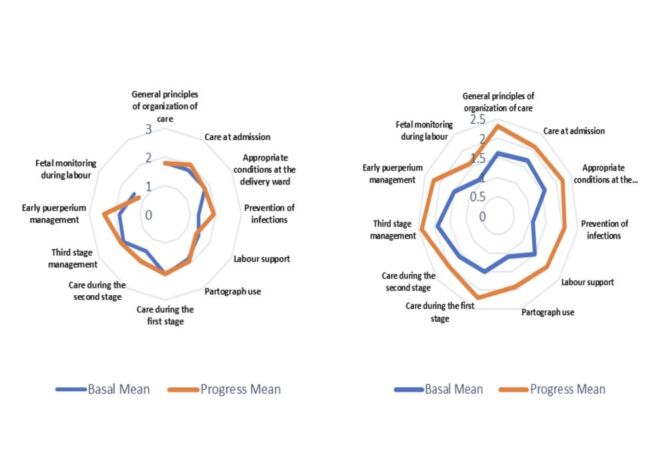
**Panel A**. Comparison of changes in normal labour and vaginal birth during baseline and end-line assessments: Kyrgyzstan. **Panel B**. Comparison of changes in normal labour and vaginal birth during baseline and end-line assessments: Tajikistan

At the end-line, the mean score improved from 1.7 to 1.9 (*P* = 0.41). Care for normal labour showed a mix of progress and stagnation. Improvements included care at admission 1.8 to 2.0 (*P* = 0.32), prevention of infections from 1.3 to 1.9 (*P* = 0.03), and early puerperium management 1.8 to 2.4 (*P* = 0.17). No change was observed in the general principles of care score 1.8 (*P* = 1.00) and conditions at the delivery ward 1.8 (*P* = 0.56). Declines were seen in labour support from 1.5 to 1.4 (*P* = 0.56) and foetal monitoring from 1.4 to 1.2 (*P* = 0.32) (**Figure 1**, Panel A).

#### Tajikistan

The baseline score 1.4 increased to 2.0 (*P* = 0.09). Improvements were seen in all areas, with the general principles of care rising from 1.6 to 2.3 (*P* = 0.01), third-stage management from 1.9 to 2.4 (*P* = 0.05), and infection prevention from 1.1 to 2.1 (*P* = 0.01). Other improvements included care during the first stage from 1.55 to 2 (*P* = 0.01), partograph use from 1.1 to 1.9 (*P* = 0.01), and foetal monitoring (1.1 to 1.6, *P* = 0.02). No declines were observed (**Figure 1**, Panel B).

### Care for caesarean section

The baseline assessment highlighted caesarean section care as a strength, with mean scores of 1.9 (1.9–2.6) in Kyrgyzstan and 1.7 (1.1–2.4) in Tajikistan, driven by 24-hour availability, low caesarean rates, and regional anaesthesia.

#### Kyrgyzstan

The end-line score increased from 1.9 to 2.1 (*P* = 0.12). Improvements were seen in surgical technique (x̄ = 1.9–2.6, *P* = 0.01) and postoperative care (x̄ = 1.7–2.1, *P* = 0.21). Policies to reduce unnecessary caesareans improved from 1.8 to 2.0 (*P* = 0.32), while readiness for emergency caesareans decreased from 2.1 to 1.9 (*P* = 0.32). No changes were found in performing caesareans based on indications (x̄ = 2.2, *P* = 1.00) or aligning with international standards (x̄ = 2.0, *P* = 1.00).

#### Tajikistan

Most areas improved, with performance per indications rising from 2.1 to 2.3 (*P* = 0.16), and alignment with international standards from 1.1 to 2.0 (*P* = 0.04). Surgical technique (x̄ = 1.4–1.8, *P* = 0.10) and postoperative care (x̄ = 1.7–1.8, *P* = 0.13) showed progress. Care after 24 hours increased from 1.9 to 2.2 (*P* = 0.10), but readiness for emergency caesareans dropped from 2.4 to 2.2 (*P* = 0.48).

### Management of maternal complications

**Figure 2:**
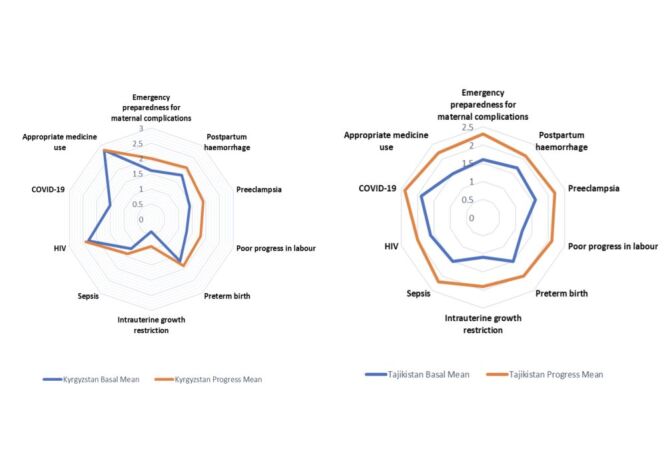
**Panel A**. Comparison of changes in the maternal complication section during baseline and end-line assessments: Kyrgyzstan. **Panel B.** Comparison of changes in the maternal complication section during baseline and end-line assessments: Tajikistan.

At baseline, the average score for managing maternal complications was 1.6 in Kyrgyzstan (range (r) = 0.4–2.8) and 1.5 in Tajikistan (r = 1.1–2.4). Final scores improved significantly to 1.9 in Kyrgyzstan (*P* = 0.01) and 2.1 in Tajikistan (*P* = 0.02) (**Figure 2**, Panels A–B).

#### Kyrgyzstan

Emergency preparedness improved from 1.6 to 2.0 (*P* = 0.14); postpartum haemorrhage management from 1.8 to 2.1 (*P* = 0.03); preeclampsia from 1.4 to 1.9 (*P* = 0.01); and prolonged labour from 1.3 to 1.8 (*P* = 0.09). Preterm birth increased from 1.7 to 1.9 (*P* = 0.11); foetal growth restriction from 0.4 to 0.9 (*P* = 0.29); and sepsis from 1.2 to 1.4 (*P* = 0.11). HIV care improved from 2.3 to 2.4 (*P* = 0.18). No change in medicine use (x̄ = 2.8; *P* = 1.00). COVID-19 data were unavailable.

#### Tajikistan

Emergency preparedness rose from 1.6 to 2.3 (*P* = 0.11); postpartum haemorrhage from 1.7 to 2.1 (*P* = 0.04); and preeclampsia from 1.6 to 2.2 (*P* = 0.67). Poor progress in labour improved from 1.2 to 2.1 (*P* = 0.01); preterm birth from 1.5 to 2.0 (*P* = 0.01); foetal growth restriction from 1.1 to 1.9 (*P* = 0.18); and sepsis from 1.5 to 2.0 (*P* = 0.31). COVID-19 preparedness increased from 1.9 to 2.4. Medicine use improved from 1.5 to 2.2 (*P* = 0.20), and HIV management from 1.6 to 2.0 (*P* = 0.01).

### Monitoring and follow-up

No change was observed in both countries; the mean score remained at 2.

### Infection prevention

#### Kyrgyzstan

**Figure 3:**
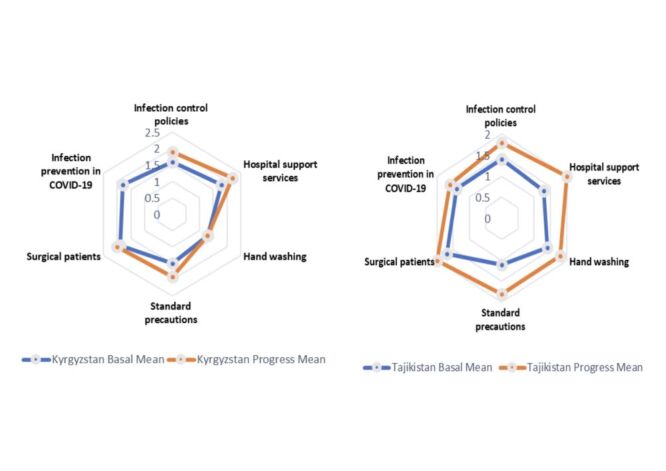
**Panel A**. Comparison of changes in infection prevention during baseline and end-line assessments: Kyrgyzstan. **Panel B**. Comparison of changes in infection prevention during baseline and end-line assessments: Tajikistan.

A tendency for improvement was observed in most variables. The infection control policies score was increased from 1.6 to 1.9 (*P* = 0.32). Hospital support services increased from 1.8 to 2.2 (*P* = 0.55), standard precautions increased from 1.5 to 1.9 (*P* = 0.20), and surgical patients' scores improved from 1.9 to 2 (*P* = 0.71). No change was observed in variable hand washing: the score remained at 1.3 (*P* = 1.00) (**Figure 3**, Panel A)

#### Tajikistan

The most variables were improved. Infection control policies score increased from 1.4 to 1.8 (*P* = 0.03). Hospital support services increased from 1.3 to 2 (*P* = 0.40), hand washing scores improved from 1.4 to 1.8 (*P* = 0.02), and standard precautions rose from 1.1 to 1.8 (*P* = 0.33). Surgical patients' score increased from 1.7 to 2 (*P* = 0.56) (**Figure 3**, Panel B)

### Guidelines and audit

Improvement was observed in both countries. In Kyrgyzstan, the score increased from 1.4 to 1.7 (*P* = 0.06). Similarly, Tajikistan also improved the quality measure, with the score rising from 1.5 to 2.0 (*P* = 0.01).

### Access to hospital care and continuity of care

Improvement was observed in both countries, with Kyrgyzstan's score improving from 1.7 to 2.0 (*P* = 0.00) and Tajikistan's from 1.5 to 2.0 (*P* = 0.01)

### Mothers’ rights

Kyrgyzstan's score shifted from 1.7 to 1.8 (*P* = 0.51) and Tajikistan’s from 1.2 to 1.7 (*P* = 0.01).

## DISCUSSION

This study demonstrates that QI interventions addressing baseline challenges have improved maternal health care in Kyrgyzstan and Tajikistan. Key domains such as labour management protocols, patient strategies, and monitoring systems showed progress. However, areas like foetal monitoring during labour, instrumental deliveries, and infection control still fall short of international standards, contributing to higher neonatal morbidity and mortality. Additionally, hyperdiagnosis of preeclampsia and suboptimal sepsis management remain concerns. Despite these advancements, further attention is needed to meet global benchmarks in maternal health. The findings underscore the importance of sustained focus on infrastructure, training, and supervision to ensure lasting improvements across all facilities.

Addressing the wide range of challenges in both countries, as described in detail in [12] and [14], requires a comprehensive approach. According to the literature, no single intervention can address all challenges, and most interventions have minor to moderate or variable effects [18,19]. This highlights the importance of multi-faceted approaches tailored to specific needs. Therefore, the initial assessment using the WHO tool was essential for understanding the areas needing improvement. The WHO Quality Assessment tool [15] provides valuable data about the quality of care. It is used in 25 countries [6,20]. Systematic assessments of the quality of care were carried out using the same tool across different regions and health systems, including a European country (Albania) and two Central Asian countries (Kazakhstan and Turkmenistan). This tool was also used in assessments and planning, creating action plans for three African countries. [20]. Our initial assessment highlighted key gaps in facility conditions, including poor infrastructure, workforce shortages, inadequate emergency care, limited partner support during birth, and mismanagement of pregnancy complications. Using specific metrics enabled us to plan targeted interventions effectively. Similar studies affirm that initial quality assessments are crucial for identifying issues, designing action plans, and establishing timelines for implementation [21]. This alignment between targeted action plans and measurable improvements is documented in Uzbekistan [22]. Da Silva et al. also observed that programmes in which providers identified a specific area for improvement before the start of intervention were instrumental in encouraging engagement and motivation [23]. Also, incorporating simple and visually appealing dashboards to track progress on indicators over time was a critical factor in the success of programmes in Uganda [24]. Following the initial quality assessment, the action plan's outlined interventions were closely aligned with the most observed improvements, as the plan set realistic and achievable targets. We have categorised the interventions that likely contributed to these measurable advancements.

### Identification and improvement of infrastructure capacity

The assessment identified that the lowest scores were linked to the health system's infrastructural capacity, often due to inefficient use of existing resources. Throughout the study, we highlighted to policymakers at both central and local levels the critical role of infrastructure in health care quality and its influence on on-site decision-making. Collaboration with regional health authorities and stakeholders led to significant improvements, including installing elevators, relocating operating theatres, and centralising oxygen supply. Similar efforts to engage policymakers in infrastructure enhancement have also been documented in QI initiatives in Uzbekistan [22]. These improvements demonstrate the tangible impact of the study's findings, particularly across most variables in Tajikistan. The relationship between infrastructure and the quality of care is well-documented in existing literature, further supporting the observed results [25].

### Developing a culture of quality

While the intervention was implemented uniformly across all hospitals, progress was observed in one facility but not in the others. Based on observations during the study, it is possible that differences in outcomes were influenced by strong leadership and consistent management, where dedicated leaders remained in place throughout the implementation process. However, further investigation would be required to establish a definitive causal link. Our approach supported effective leadership by offering authoritative guidance and a comprehensive framework for change. We emphasised fostering a culture of improvement over blame. Local health teams were encouraged to start with simple interventions for early wins, gradually building management skills to tackle more complex challenges. Committee members were trained in QI tools to track progress, manage quality indicators, and identify barriers. As part of the initiative, teams were encouraged to share experiences and collaborate within networks, promoting personal growth, stakeholder recognition, and a more profound commitment to QI.

Necocheat et al. effectively used a similar method and showed improved provider performance using exemplary sharing [26]. Several studies demonstrated that this method creates ownership of any introduced QI programme [18,27]. A successful example of this approach was in Zambia [28]. Over five years, the country's capacity for QI has improved. Yemen is another country that has developed a culture that values QI [29]. The impact of successful QI leaders is evident from a similar study from Indonesia [30]. Moreover, local decision-makers can ensure the alignment of QI projects with organisational strategy, translate strategic priorities into actionable tasks, and delegate responsibilities [31].

### Enabling a competent and motivated health workforce

#### Effective perinatal care training

The assessment revealed gaps in knowledge and adherence to international recommendations, leading to suboptimal care. A capacity-building programme addressed these gaps, focusing on labour, complications, and infection control, with on-site training to improve skills and protocol compliance. Over 500 providers in each country were trained, resulting in increased assisted vaginal births, better labour monitoring, and improved management of postpartum haemorrhage and preeclampsia. This approach proved effective in strengthening maternal and newborn health services. In Malawi, Bangladesh, Pakistan, and Guatemala, the training and close supervision of traditional birth attendants demonstrated the potential to reduce harmful practices during delivery and childbirth while improving overall maternal health outcomes [32].

#### Supportive supervision

The initial assessment identified limited professional development opportunities and a punitive supervision system focused on fault-finding as contributors of the poor maternal care quality in both countries. To address this, a supportive supervision approach was integrated into the existing framework, involving training supervisors and promising professionals, on-site capacity building, and international consultancy support. This approach not only enhanced hospital capacity but also reduced the punitive nature of the traditional system. Regular follow-ups demonstrated that supportive supervision improved clinical competencies and performance in maternity care settings. Other studies also consider it an optimal QI approach, as it replaces traditional supervision methods with a focus on facilitation rather than inspection, as seen in Kyrgyzstan [33].

#### Clinical guideline implementations

The changes can also be attributed to updating and creating clinical guidelines based on the latest evidence. Research shows that implementing standards improves maternal care. Implementing maternal quality of care standards in Bangladesh, Ghana, and Tanzania demonstrated the adaptation of respectful maternity care. [34] Other studies also emphasise the potential of evidence-based guidelines and the potential for improving the quality of care and maternal care outcomes [35,36]. All institutions now have updated national protocols for managing vaginal birth and obstetric complications, addressing conditions like preeclampsia, haemorrhage, and obstructed labour. While barriers like resistance to change and inconsistent compliance remain, the availability of these guidelines and related training marks a critical step toward more standardised, effective care.

The study highlighted areas that needed improvement, such as foetal monitoring, sepsis management, and infection control, while emphasising the importance of refining quality improvement initiatives for sustained progress. Key recommendations include:

1) ongoing professional development

2) regular follow-up assessments

3) more significant involvement of frontline workers in intervention design

4) strengthening infrastructure and resources.

### Limitations and strengths

This study has several limitations. The 24-month duration may not fully capture the long-term sustainability of improvements or maternal and neonatal health outcomes. The lack of a comparator group limits our ability to attribute changes directly to the quality improvement initiatives. Additionally, the intervention did not address resource limitations, such as staffing, funding, and supplies, which could impact the sustainability of improvements. Nevertheless, the study’s strengths, including using a standardised tool, a targeted assessment approach, and trained assessors, support the validity of the findings.

## CONCLUSIONS

The study demonstrated that implementing planned interventions, coupled with close monitoring and efficient quality-enhancing methods, improved the quality of care in some areas across most hospitals in two lower-middle-income countries within a short period. However, some domains showed little to no progress. Significant challenges persist, with many domains still falling below established standards of care. While the findings suggest a positive trend, the lack of a control group and potential external influences preclude definitive conclusions. Still, consistent patterns in key indicators suggest a possible contribution from the interventions. The sustainability of these changes over time remains uncertain. Continued efforts are necessary to maintain and extend these gains across additional health care facilities. Given the mixed results, scaling these interventions should be approached cautiously, focusing on addressing remaining gaps and ensuring long-term impact.
